# The role and controversy of pelvic lymph node dissection in prostate cancer treatment: a focused review

**DOI:** 10.1186/s12957-024-03344-2

**Published:** 2024-02-26

**Authors:** Baonan Dong, Hui Zhan, Ting Luan, Jiansong Wang

**Affiliations:** Urology Surgery Department, The Second Affiliated Hospital of Kunming Medical University, 243 Dianmian Avenue, Wuhua District, Kunming, 650101 Yunnan China

**Keywords:** Pelvic lymph node dissection, Radical prostatectomy, Oncological outcomes

## Abstract

Pelvic lymph node dissection (PLND) is commonly performed alongside radical prostatectomy. Its primary objective is to determine the lymphatic staging of prostate tumors by removing lymph nodes involved in lymphatic drainage. This aids in guiding subsequent treatment and removing metastatic foci, potentially offering significant therapeutic benefits. Despite varying recommendations from clinical practice guidelines across countries, the actual implementation of PLND is inconsistent, partly due to debates over its therapeutic value. While high-quality evidence supporting the superiority of PLND in oncological outcomes is lacking, its role in increasing surgical time and risk of complications is well-recognized. Despite these concerns, PLND remains the gold standard for lymph node staging in prostate cancer, providing invaluable staging information unattainable by other techniques. This article reviews PLND's scope, guideline perspectives, implementation status, oncologic and non-oncologic outcomes, alternatives, and future research needs.

## Lymphatic drainage in prostate cancer

The male pelvic region's lymph nodes can be categorized into five distinct groups based on their placement, specifically the paraaortic nodal group, the common Iliac nodal group, the internal Iliac nodal group, the external Iliac nodal group, and the inguinal lymph nodal group. The various clusters of lymph nodes are linked together to create a lymphatic drainage system in the pelvic area. The male pelvic tissues and organs can be drained lymphatically through three primary routes: the superficial inguinal pathway, the pelvic pathway, and the paraaortic pathway. The pelvic pathway, comprising the common iliac group, the internal iliac group, and the external iliac group, serves as the primary lymphatic drainage pathway for both the bladder and prostate [1]. Lymph nodes situated near the iliac vessels are typically the initial targets of lymph node metastases originating from prostate cancer, with the internal iliac region exhibiting the highest vulnerability, followed by the external iliac region, the obturator region, the common iliac region, and the presacral region [2, 3]. Sentinel lymph nodes are typically seen as the main source of lymphatic drainage from tissues and organs, and they are the most probable spot in the lymphatic drainage chain where tumors are likely to spread first. The Fossa Marcille, situated within the male pelvis, serves as a crucial anatomical framework [4, 5], encompassing lymph nodes from the external iliac, obturator, and internal iliac regions that collectively contribute to its composition. The presence of positive lymph nodes in the fossa Marcille in patients with prostate cancer is often accompanied by negative lymph nodes in other areas [6], indicating a strong association between lymph node invasion in this region and the occurrence of multiple lymph node metastases in other regions [5], implying a high likelihood of sentinel lymph nodes for prostate cancer in the fossa Marcille, which aligns with the lymphatic drainage attributes of prostate cancer. By employing the technique of contrast agent localization, pertinent research indicated that the sentinel lymph nodes of prostate cancer were predominantly clustered in the obturator region (30%-40%), the external iliac region (19%-34%), and the internal iliac region (17%-33%) [7, 8], while subsequent investigations unveiled that the sentinel lymph nodes were predominantly positioned at the intersection of the internal iliac and external iliac vessels, along with the distal extremity of the internal iliac vessels [7], By identifying the sentinel lymph nodes, we can ascertain the severity of the illness, diminish the trauma caused by surgery, and give advice for treatment choices and prognostic evaluation [2].

While prostate cancer exhibits the aforementioned drainage properties, the actual drainage scenario may become more intricate when lymph node metastasis occurs. In light of the potential for lymphatic dissemination in prostate cancer cells, urologists commonly recommend PLND for localized prostate cancer patients exhibiting an elevated risk of lymph node infiltration. In instances where pelvic lymph node metastasis has been conclusively identified, conventional therapeutic interventions such as chemotherapy, radiotherapy, and immunotherapy are generally prioritized. Salvage PLND, proposed as an alternative treatment in select medical centers, remains a subject of ongoing debate regarding its therapeutic efficacy. In summary, the primary objectives of the PLND are to elucidate the classification of the tumor lymph nodes and eliminate potential lymph node metastatic lesions, with the aim of attaining potential therapeutic advantages and enhancing the patient's prognosis [9, 10].

## The excision range of PLND

In general, pelvic lymph node dissection (PLND) is a surgical intervention that eliminates lymph nodes from different areas along the iliac vascular distribution, taking into account the manner in which lymphatic drainage occurs in prostate cancer. PLND can be broken down into distinct subcategories depending on the particular regions examined. Nevertheless, there is a lack of consensus among academics regarding the extent of excision within the PLND subgroup. A quadratic division of PLND has been suggested by certain scholars [11]: (1) limited pelvic lymph node dissection (lPLND): external iliac region + obturator region; (2) standard pelvic lymph node dissection (sPLND): lPLND + internal iliac region; (3) extended pelvic lymph node dissection (ePLND): sPLND + common iliac region; (4) super-extended pelvic lymph node dissection ( sePLND): ePLND + presacral region. However, there are also scholars who have proposed a trichotomy of PLND [3]: lPLND (external iliac region + obturator region), ePLND (lPLND + internal iliac region), and sePLND (ePLND + common iliac region + presacral region).

## The indications of PLND

The academic community continues to hold differing opinions on the timing of treatment and patient selection for PLND. Proponents of PLND argue that this procedure not only assesses the tumor's lymph node staging but also eliminates metastatic lesions from the lymph nodes for therapeutic purposes [12, 13], Conversely, opponents of PLND argue that it can result in extended surgical duration and increased complications, and that the decision to undergo PLND does not impact the patient's postoperative oncological prognosis. Various nations and territories also offer distinct suggestions for PLND.

According to the European Association of Urology (EAU) guidelines, PLND is advised for patients with intermediate-risk prostate cancer who have a high likelihood of lymph node invasion (with cut-off values ranging from 5 to 7%), as evaluated by Briganti nomogram, as well as for those with high-risk prostate cancer. It is advisable to use ePLND, which includes the external iliac, obturator, internal iliac, and common iliac regions [14]. According to the American Urological Association (AUA) guidelines, PLND can offer crucial insights into lymph node staging and contribute to the formulation of a post-surgical treatment strategy, yet it does not yield substantial advantages in terms of oncological outcomes. Hence, it is advisable to evaluate the likelihood of lymph node invasion through pertinent nomograms prior to deciding whether to carry out PLND. It is advisable to use ePLND, which includes the external iliac, obturator, and internal iliac regions [15]. In accordance with the National Comprehensive Cancer Network (NCCN) guidelines, PLND assists in elucidating the staging of tumorous lymph nodes and eradicating microscopic lymphatic metastatic lesions. Hence, it is advisable to utilize nomograms for evaluating the likelihood of lymph node infiltration in patients and to conduct ePLND (encompassing the external iliac, obturator, and internal iliac regions) after thoroughly evaluating surgical duration, potential surgical complications, and other variables [16]. According to China's prostate cancer treatment guidelines, there is inadequate evidence to substantiate the positive impact of PLND on on oncological outcomes, nevertheless, PLND can elucidate lymph node staging and offer prognosis guidance. Hence, it is advisable for patients diagnosed with high-risk prostate cancer and those identified as intermediate-risk with an elevated risk of lymph node invasion based on the Memorial Sloan Kettering Cancer Center (MSKCC) nomogram (with a cut-off value of 5%) to undergo ePLND therapy (encompassing external iliac, obturator, and internal iliac regions) (Table 1).

**Table 1:** Summary of different recommendations for PLND in different guidelines

Guideline	Risk group	Assessment method	Threshold	Recommand PLND	PLND template	Recommendation Strength
EAU	Intermediate	Briganti nomogram	5%	ePLND	Obturator Internal iliac External iliac Common iliac	Strong
	High	/	/			Strong
AUA	NA	Nomogram	NA	ePLND	Obturator Internal iliac External iliac	Moderate
NCCN	Intermediate (Favorable & Unfavorable) High Very high	Nomogram	2%-7%	ePLND	Obturator Internal iliac External iliac	2A
China	Intermediate	MSKCC nomogram	5%	ePLND	Obturator Internal iliac External iliac	/
	High	/	/			

*Abbreviations*: *PLND* pelvic lymph node dissection, *ePLND* extended PLND, *EAU* European Association of Urology, *AUA* American Urological Association, *NCCN* National Comprehensive Cancer Network, *MSKCC* Memorial Sloan Kettering Cancer Center

After conducting a thorough analysis of multiple guidelines from various countries and regions, it has been determined that PLND is generally not advisable for patients with low-risk prostate cancer, whereas ePLND is recommended for patients with intermediate-risk prostate cancer who have a higher likelihood of lymph node invasion, as indicated by nomograms such as MSKCC and Briganti. In addition to patients with a high-risk prostate. In relation to the range of lymph node dissection, despite the recommendation of various guidelines for ePLND, the EAU's recommended range of ePLND includes the common iliac region alongside the range of ePLND dissection (external iliac + obturator + internal iliac) recommended by the AUA, NCCN, and China guidelines. It is crucial to delve deeper into the potential variations in lymph node staging and oncological outcomes associated with ePLND of such varying ranges.

## The current status of PLND

Numerous guidelines mentioned earlier advocate for the provision of PLND to all patients diagnosed with intermediate- and high-risk prostate cancer, on the condition that the necessary criteria are fulfilled; however, the practical implementation rate of PLND remains relatively inadequate. A study conducted in the UK examined high-risk prostate cancer patients who received surgical treatment in accordance with the EAU guidelines. Among the 3091 high-risk prostate cancer patients, PLND treatment was administered to 66%, while 62.4% either did not undergo PLND or solely underwent obturator lymph node dissection. Furthermore, out of the 66% who received PLND treatment, a mere 56.8% opted for ePLND treatment, with less than one-third of ePLND being performed in its entirety. The findings of this study propose that the diminished rate of PLND performance could be attributed to the abundance of perioperative complications associated with PLND, without any notable improvement in survival outcomes [17]. In accordance with the German S3 guidelines, a multicenter study conducted in Germany and Austria demonstrated an impressive overall implementation rate of 97.2% for PLND [18]. A North American study assessed the adherence rate to guideline-recommended PLND among patients with prostate cancer in North America. The study findings indicated that, as per the NCCN guidelines and the D'Amico risk stratification scheme, the recommended rates for PLND were 63.3% and 64.9% for patients, and 68.8% and 69.1% for those who actually underwent PLND, respectively, indicating a persistently low percentage, despite an increase compared to previous records. The study determined that when making clinical decisions, it is important to take into account not only guideline recommendations but also the clinical characteristics of patients. Patients with a low likelihood of lymphatic metastasis but unfavorable traits of prostate cancer (such as late clinical-stage, high Gleason score, and a significant proportion of positive biopsies) should be considered for PLND, while those with a high risk of lymphatic metastasis but lacking unfavorable characteristics of prostate cancer can be deemed to have PLND disregarded [19].

Taking into account the escalating pattern of lymph node invasion in prostate cancer patients annually [20], the guidelines advocate for the adoption of PLND in screened prostate cancer patients. Nevertheless, the diverse cognitive viewpoints regarding PLND across various centers, surgical methodologies, and other variables have resulted in significant fluctuations in the practical implementation of PLND in clinical settings.

## The oncological outcomes of PLND

The variation in PLND implementation rates can be attributed, in part, to the absence of a consistent perspective on the significance of PLND in the clinical context. The question of whether PLND should be conducted, the appropriate level of PLND to be administered, and the actual benefits it provides to patients with prostate cancer has sparked controversy.

### Should PLND be implemented?

Many investigations have been carried out to determine if PLND should be put into practice. Badani et al. conducted a retrospective analysis on the survival rates of patients with pT3b prostate cancer who underwent RP, revealing that the risk of postoperative biochemical recurrence was comparable between the two patient groups who did not undergo PLND and those who did; however, in patients who underwent PLND, the augmentation in the number of dissected lymph nodes did not significantly diminish the probability of biochemical recurrence [21]. Preisser et al.’s study discovered that in a cohort of patients diagnosed with intermediate-risk and high-risk prostate cancer who were administered ePLND compared to those who were not, ePLND effectively furnished data regarding the classification of the tumor lymph nodes. Nevertheless, there was no notable disparity between the two cohorts in regards to biochemical recurrence-free survival, metastasis-free survival, and tumour-specific survival [22, 23]. Tomisaki et al's study arrived at a comparable finding: individuals with limited prostate cancer who underwent PLND exhibited no notable disparity in oncological outcomes in comparison to those who did not undergo PLND, and patients who did not undergo PLND circumvented the accompanying surgical hazards and complications [24]. Kodiyan et al. collated information from the US National Cancer Database in accordance with the NCCN guidelines and categorized patients into two categories: those with positive attributes of prostate cancer and those with negative traits of prostate cancer. While the acceptance or rejection of PLND did not significantly impact survival in the group exhibiting favorable traits of prostate cancer, a correlation was observed between the implementation of PLND and improved survival in the group with unfavorable characteristics of prostate cancer [25]. According to the US National Cancer Database, Sood et al. conducting ePLND yields a notable survival benefit for individuals with lymph node metastases (pN1) in contrast to the absence of PLND or ePLND, and for every extra lymph node examined, a patient's tumor-specific survival rate can be enhanced by 7% [26].

### Optimal excision range of PLND

Besides the decision of whether or not to receive PLND, the selection of the appropriate level of PLND to be granted is a subject that warrants thorough exploration. Lestingi et al. conducted a phase III randomized controlled clinical trial at a single center, with a prospective design. I examined if there was any disparity in oncological results among 300 intermediate- to high-risk prostate patients who underwent radical prostatectomy with ePLND (including external iliac, obturator, internal iliac, common iliac and presacral regions) and lPLND (only the presacral region) to determine if there was any variation in oncological outcomes. Initial findings indicate that while ePLND offers more conclusive data regarding tumor lymph node staging, there is no statistically significant disparity between the two in relation to 5-year biochemical recurrence-free survival, metastasis-free survival, or tumour-specific survival. Subgroup analyses indicate that ePLND has the potential to be advantageous for patients with high-grade prostate cancer in relation to biochemical recurrence-free survival, although further studies are required to validate these subgroup analyses [27, 28]. Touijer et al. devised a controlled trial with a single center and randomization. A comparison was made between the oncological results of lPLND (external iliac region only) and ePLND (external iliac + obturator + internal iliac) in 1440 patients with prostate cancer. The findings indicated that the disparities in the quantity of dissected lymph nodes and the percentage of positive lymph nodes were negligible between the two groups, and that ePLND did not diminish the likelihood of biochemical recurrence in patients [29]. Wettstein et al. conducted a retrospective study involving two centers, focusing on a cohort. It was proven that ePLND effectively decreased the likelihood of biochemical recurrence and the necessity for supplementary treatment post-surgery by 25%-31% in contrast to non-ePLND [30]. Preisser et aI. performed a retrospective analysis utilizing the SEER database to assess the survival rates of individuals with prostate cancer who had undergone PLND. Patients with no lymph node invasion and postoperative pathology exhibited a decreased tumor-specific mortality rate following a more extensive PLND, in contrast to patients with less extensive PLND. Additionally, each additional lymph node removed by PLND resulted in a reduction of approximately 4.5% in tumour-specific mortality, which was unfavorable for the prostate cancer group [31].

It is evident from the aforementioned studies that there are conflicting findings regarding the potential oncological benefits of PLND. Despite certain studies suggesting the potential oncological benefits of PLND in specific patient groups, such as those with unfavorable characteristics of prostate cancer, these studies have predominantly focused on retrospective research, limited prospective studies, bias in patient selection for PLND, non-standardized templates for PLND, and absence of a comprehensive description of the specific dissection range of PLND, among other factors (Table 2 [27, 29, 30, 32–35]). In order to bridge the gap between studies, a more effective study design should be implemented in the future to ensure more dependable findings.

**Table 2:** Summary of several studies comparing oncological outcomes of PLND in RP

Study&Type	Study period	Cohort	Cohort size	Risk category	Pathological stage	Gleason score	ISUP grade	Positive surgical margin	PLND template	Removed LNs (median)	Positive LNs rate	Outcome measure	Follow-up duration (median)	Oncological outcomes
Fergany et al. [32] Retrospective	1986- 1999	RRP noPLND	203	NA	≥ pT3b(1.0%)	≤ 6(61%) ≥ 7(39%)	NA	NA	/	/	/	Biochemical failure, Biochemical relapse-free survival(BCRFS)	38mo	Biochemical failure: 7.0%, *n* = 40(33 in PLND group + 7 in noPLND group) 4-yr BCRFS: 97%(noPLND) vs 91%(PLND) (*p* = 0.16) PLND or noPLND was not independent predictor of BCR
		RRP PLND	372	NA	≥ pT3b(4.0%)	≤ 6(60%) ≥ 7(40%)	NA	NA	NA	NA	1.6%			
Furubayashi et al. [33] Retrospective	1998- 2013	RP sPLND	247	NA	≤ pT2(59.5%) ≥ pT3(40.5%)	≤ 7(76.5%) > 8(23.5%)	NA	23.5%	Obturator Internal iliac	13	NA	PSA failure	53.7mo	PSA recurrence: sPLND(17.8%) vs ePLND + mePLND(13.9%)(*p* = 0.36) Multivariate analysis: The range of PLND(standard vs.extended) is significantly associated with PSA failure(HR 2.099, [95%CI 1.140–4.099], *p* = 0.0164)
		RP ePLND	78	NA	≤ pT2(55.1%) ≥ pT3(44.9%)	≤ 7(70.5%) > 8(29.5%)	NA	17.9%	Obturator Internal iliac External iliac	19	NA			
		RP mePLND	23	NA	≤ pT2(60.9%) ≥ pT3(39.1%)	≤ 7(73.9%) > 8(26.1%)	NA	13.1%	Obturator Internal iliac External iliac Common iliac	19	NA			
Wettstein et al. [30] Retrospective	2006- 2016	RP ePLND	368	NA	≥ pT3(39.1%)	NA	1(4.9%) 2(43.5%) 3(26.9%) 4(7.1%) 5(17.7%)	35.6%	Obturator Internal iliac External iliac Common iliac	NA	10.3%	Time to biochemical recurrence(BCR) or secondary therapy	3.5 yr	BCR or secondary therapy: ePLND 22.6% vs nePLND 22.7% Survival analysis: ePLND(weighted HR: 0.75[95%CI 0.56–0.99], *p* = 0.044) Casual mediation analysis: protective direct effect of 0.69(0.63–0.75)
		RP nePLND	640	NA	≥ pT3(55.0%)	NA	1(7.3%) 2(52.7%) 3(25.9%) 4(6.6%) 5(7.5%)	25.9%	NA	NA	5.2%		2.2 yr	
Iwamura et al. [34] Retrospective	2010- 2020	RP lPLND	283	High(100%)	pT0(10.2%) pT2(59.4%) pT3(30.4%)	No residual tumor(10.2%) 6(0.4%) 7(4.3%) 8(6.7%) 9–10(78.4%)	NA	6.0%	Obturator	4	0.8%	BCRFS	77mo	3 yr BCRFS: 89.1%(lPLND) vs 86.0%(nonPLND), *p* = 0.516 5 yr BCRFS: 84.1%(lPLND) vs 82.0%(nonPLND), *p* = 0.516 COX regression analysis: BCRFS is not significantly diferrence between two groups(HR 1.44, *p* = 0.469)
		RP nonPLND	233		pT0(11.2%) pT2(62.7%) pT3(26.2%)	No residual tumor(11.2%) 6(0.9%) 7(27.0%) 8(0.9%) 9–10(60.1%)	NA	11.6%	/	/	/		34mo	
Touijer et al. [29] RCT	2011- 2017	RP ePLND	740	NA	≥ pT3(49%)	NA	1(7.3%) 2(53%) 3(24%) 4(16%) Unknown	NA	Obturator Internal iliac External iliac	14	14.0%	BCR	3.1 yr	BCR rate: No difference (HR 1.04, [95%CI 0.93–1.15], *p* = 0.5 for lPLND as the reference group)
		RP lPLND	700	NA	≥ pT3(54%)	NA	1(5.9%) 2(58%) 3(23%) 4(12%) Unknown	NA	External iliac	12	12.0%			
Namiki et al. [35] Retrospective	2012- 2021	RARP nonPLND	605	Low(16.0%) Intermediate(70.4%) High(13.6%)	pT2(22.0%) pT3(78.0%)	NA	1(9.1%) 2(51.0%) 3(27.6%) 4(8.3%) 5(4.0%)	20.7%	/	/	/	BCRFS	17.2mo	3 yr BCRFS: nonPLND 93.7% vs PLND 91.5%(*p* = 0.855) BCRFS: No difference between PLND and nonPLND in the all-risk group
		RARP PLND	605	Low(13.9%) Intermediate(70.2%) High(15.9%)	pT2(20.7%) pT3(79.3%)	NA	1(10.2%) 2(50.5%) 3(27.6%) 4(7.1%) 5(4.6%)	19.8%	Limited (Obturator) Extended(NA)	5	1.0%		24.7mo	
Lestingi et al. [27] Prospective	2012- 2016	RP ePLND	150	Intermediate(62%) High(38%)	pT0(0) pT2(41%) pT3a(45%) pT3b(14%) pT4(0.7%)	6(2.7%) 7(85%) 8(1.3%) 9–10(11%)	NA	44.0%	Obturator Internal iliac External iliac Common iliac Presacral	17	17.0%	BCRFS MFS(Metastasis-free survival) CSS(Cancer-specific survival)	53.9mo	Median BCRFS: not reached(ePLND) vs 61.4mo(lPLND)(HR 0.91, [95%CI 0.63–1.32], *p* = 0.6) Median MFS: not reached in either group(HR 0.57,[ 95%CI 0.17–1.8], *p* = 0.3) CSS: NA
		RP lPLND	150	Intermediate(63%) High(37%)	pT0(0.7%) pT2(38%) pT3a(43%) pT3b(18%) pT4(0.7%)	6(4%) 7(80%) 8(0.7%) 9–10(15%)	NA	37.0%	Obturator	3	3.4%			

*Abbreviations***:** *RP* radical prostatectomy, *RRP* retropubic radical prostatectomy, *RARP* robotic-assisted radical prostatectomy, *PLND* pelvic lymph node dissection, *lPLND* limited PLND, *sPLND* standard PLND, *ePLND* extended PLND, *mePLND* more extended PLND, *nePLND* non extended PLND, *BCR* biochemical recurrence, *BCRFS* biochemical recurrence(relapse)-free survival, *HR* hazard ratio, *CI* confidence interval, *NA* not applicable, *yr* years, *mo* months, *MFS* metastasis-free survival, *CSS* cancer-specific survival

## The non-oncological outcomes of PLND

### Lymph node staging

Regarded as the epitome of lymph node staging, PLND offers accurate data on tumor staging in individuals with prostate cancer, aiding in the evaluation of the patient's state and offering valuable insights for their prognosis. Notwithstanding the utilization of medical imaging technologies like CT, MRI, PET-CT, etc. The utilization of relevant clinical parameters to assess lymph node staging has proven to be effective alternatives; however, the actual pathological information of lymph nodes obtained from these alternatives remains limited when compared to PLND, and there is still potential for further enhancement in determining lymph node staging [36].

### Surgical complications

As surgical techniques have progressed, PLND has gone from the early days of open surgery to the current era of laparoscopy and robot-assisted laparoscopy. While there has been a decline in the complication rate of PLND in comparison to previous instances [37], there are still certain complications that necessitate attention. The typical complications associated with PLND encompass lymphocysts, harm to pelvic tissues and organs (such as vessels, obturator nerves, bladder, and rectum), venous thrombosis in the lower extremities, and sexual dysfunction. The disruption of lymphatic drainage pathways in the pelvis caused by PLND makes lymphocysts the prevailing complication. Based on the available literature, the likelihood of lymphocysts following PLND varies between 1.3% and 25% [38–40], and their presence is not solely attributed to the enlargement of PLND excision [38, 41], but also to advanced age, elevated BMI (Body Mass Index), and a past occurrence of peripheral vascular/lymphatic lesions [41–44]. To reduce the number of lymphocysts, preventive measures such as constructing a peritoneal flap [45, 46]、peritoneal opening [47, 48], elastic clamping of lymphatic vessels [39], bipolar electrocoagulation of the trauma, and spraying of haemostatic powder [49] can be taken during operation. Nevertheless, lymphocysts do not consistently manifest symptoms, and interventions are typically limited to symptomatic lymphocysts (such as excessive abdominal-pelvic effusion or secondary infections). Subsequent to PLND, a range of medical interventions including lymphangiography, lymphatic embolisation [50], prolonged pelvic drainage, reoperation for exploration, or puncture and drainage may be undertaken [37]. In contrast to lymphocysts, complications like pelvic tissue and organ damage, lower extremity venous thrombosis, and sexual dysfunction are uncommon and can be prevented through meticulous anatomical separation during PLND and improved perioperative patient care [37, 38].

## The alternatives of PLND

Despite PLND being widely regarded as the gold standard for lymph node staging in prostate cancer patients, numerous studies have strived to investigate the utilization of non-PLND techniques in assessing lymph node invasion and preventing superfluous PLND, considering the ongoing controversy surrounding the oncological advantages of PLND and the potential for extended surgical duration and related complications. In the past, a great deal of research has been conducted to evaluate the clinicopathological features of prostate cancer patients, taking into account clinical factors related to the disease. A range of prediction models have been created to forecast the risk of lymph node invasion, with Briganti, MSKCC, Cagiannos, Formulas, Zumsteg, etc. being the most popular ones [51–53]. Additionally, there are numerous studies that have been externally validated to determine their predictive power in various populations [52, 54–56]. EAU, AUA, NCCN, and other guidelines suggest the use of these prediction models with clinical parameters, which can be adjusted to achieve the best possible results for different patient groups by changing their cut-off values, a widely used method for assessing lymph node invasion.

CT and MRI can be used to evaluate prostate cancer patients before surgery to determine the condition of the primary tumor lesion, however, relying solely on CT or MRI to acquire data on pelvic lymph nodes is quite restricted. Research data indicates that CT scans have a sensitivity of 8.9% and a specificity of 98.3% when identifying positive pelvic lymph nodes, whereas MRI scans have a sensitivity of 14.3% and a specificity of 98.8% [57], indicating that relying solely on CT or MRI to decide whether to proceed with PLND is not dependable. The current recommendation for predicting pelvic lymph node invasion in clinical practice is to use multiparametric MRI in combination with tumour-related clinical parameters. To provide an instance, Brembilla et al. conducted a predictive model, which combined tumour volume (mrV) and T-stage (mrT) from MRI, along with clinical parameters like preoperative PSA and major Gleason scores, to predict pelvic lymph node invasion, and its AUC value is 0.956. By setting the cut-off values of mrT3 and mrV ≥ 1cc for PLND, it was possible to decrease 55.4% of unnecessary PLNDs and only 4.3% of patients with lymph node invasion were overlooked [58]. Gandaglia et al. developed a predictive model to accurately predict pelvic lymph node invasion by utilizing the MRI index lesion's maximum diameter, the pathology grading of the MRI-directed biopsy region, and various clinical parameters such as PSA, clinical stage, and the percentage of biopsy-positive needle counts, resulting in a remarkable 60% reduction in ePLNDs and a mere 1.6% ignorance of lymph node metastases among patients with a cut-off value of 7% [59]. Many clinical prediction models that combine multiparametric MRI and other relevant clinical parameters to predict pelvic lymph node invasion have shown a significant improvement in predictive efficacy when compared to previous models that only incorporate clinical parameters [60–63]. PET-CT, due to its remarkable ability to visualize tumour foci, has been extensively employed to identify prostate cancer lymph node invasion and can direct the removal of positive lymph nodes during surgery [64]. As an illustration, ^68^Ga PSMA PET-CT can be employed to guide the excision of positive lymph nodes and prevent the elimination of uninvolved lymph nodes. This method exhibits a 67% sensitivity, 100% specificity, 100% positive predictive value, and 90% negative predictive value in identifying positive lymph nodes at the individual patient level [65].

In the present scenario, where PLND is regarded as the gold standard for assessing lymph node invasion, various techniques for evaluating lymph node invasion have arisen collectively. These techniques have exhibited favorable prognostic outcomes across various cohorts of prostate cancer patients, yet they necessitate additional investigation and investigation in terms of precisely acquiring lymph node pathology data, forecasting tumor survival prognosis, ensuring safety, and assessing cost in comparison to PLND.

## Conclusion and outlook

Pelvic lymph node dissection is a surgical procedure that removes pelvic lymph nodes based on the characteristics of pelvic lymphatic drainage in order to clarify the staging of the tumour lymph nodes and obtain potential therapeutic benefits. Numerous national and regional guidelines endorse this procedure as the gold standard for lymph node staging of prostate cancer, yet there remains a certain level of debate regarding its potential oncological advantages. As medical technology advances, there are now a variety of techniques that integrate radiology and clinical parameters to evaluate the danger of lymphatic infiltration in individuals with prostate cancer, and the accuracy of these approaches has been verified in various patient cohorts, however, their actual efficacy in comparison to PLND must be further verified in a broader patient group.

When conducting future research on PLND, it is imperative to prioritize the following crucial aspects: establishing a uniform categorization of PLND subgroups, determining the most suitable excision range for PLND, enhancing the predictive precision of diverse predictive models, elucidating the potential oncological advantages of PLND for prostate cancer patients, and exploring alternative non-PLND treatment options for positive lymph nodes.

## Data Availability

No datasets were generated or analysed during the current study.

## Abbreviations

RP: Radical prostatectomy
RARP: Robotic-assisted radical prostatectomy
PLND: Pelvic lymph node dissection
lPLND: Limited PLND
sPLND: Standard PLND
ePLND: Extended PLND
sePLND: Super extended PLND
EAU: European Association of Urology
AUA: American Urological Association
NCCN: National Comprehensive Cancer Network
MSKCC: Memorial Sloan Kettering Cancer Center
BMI: Body Mass Index
PSA: Prostate specific Antigen
